# *Bacillus subtilis* PB6 based probiotic supplementation plays a role in the recovery after the necrotic enteritis challenge

**DOI:** 10.1371/journal.pone.0232781

**Published:** 2020-06-18

**Authors:** Mashael R. Aljumaah, Manal M. Alkhulaifi, Alaeldein M. Abudabos, Riyadh S. Aljumaah, Asma N. Alsaleh, Dragana Stanley

**Affiliations:** 1 Department of Botany and Microbiology, College of Science, King Saud University, Riyadh, Saudi Arabia; 2 Department of Animal Production, College of Food and Agriculture Sciences, King Saud University, Riyadh, Saudi Arabia; 3 Institute for Future Farming Systems, Central Queensland University, Rockhampton, Queensland, Australia; Tokat Gaziosmanpasa University, TURKEY

## Abstract

In poultry production, birds are raised under intensive conditions, which can enable rapid spread of infections, with *Clostridium perfringens*-caused necrotic enteritis (NE) being one of the most devastating for the industry. The current investigation was conducted to evaluate the effectiveness of *Bacillus subtilis* PB6 probiotic supplementation on bird’s post NE recovery, based on chicken performance, cecal microbiota composition, ileum histomorphometric measurements, and short-chain fatty acid production in the cecum of the birds that were challenged with NE mid-production. Birds were split into four groups, including a negative control, positive control challenged with *C*. *perfringens*, group supplemented with *B*. *subtilis* probiotic, and NE challenged birds supplemented with *B*. *subtilis* probiotic. Following NE challenge birds were allowed to reach the end of production time at 40 days, and samples were collected to estimate if probiotic supplementation resulted in better post-NE recovery. Intestinal lesion score across the duodenum, jejunum, and ileum indicated that at the end of production timeline NE challenged birds supplemented with *B*. *subtilis* probiotic had lower intestinal lesion scores compared to NE challenged birds without probiotic supplementation implying improved recovery. Probiotic supplementation improved performance of NE challenged birds only in the post-NE recovery stage. NE challenged birds had a significant increase in cecal propionic acid, which was not observed in NE challenged birds supplemented with *B*.*subtilus*. Both *B*. *subtilis* supplemented groups (challenged and unchanged) were characterized by a significant rise in cecal acetic and butyric acid. Our results demonstrate that *B*. *subtilis* supplementation can assist the birds in dealing with NE outbreak and long term recovery.

## Introduction

Decades of research and genetic selection have resulted in today’s broiler chicken consuming three times less food to reach the required market weight compared to broilers commonly raised in the 1950s [1]. The poultry industry is the fastest-growing meat production industry (USDA ERS), propelled by a relatively low cost of poultry meat and eggs compared to other sources of animal protein. To reach the substantial consumer demand for poultry products, birds are raised under intensive farming conditions with up to 100,000 birds per flock, facilitating the swift spread of infections [2] which remains the major issue for the rapidly growing industry.

Since the early 1950s when the growth-promoting effects of antibiotics use in animal feed were first used, antibiotic growth promoters (AGPs) were regularly added to feed to enhance growth, reduce the cost of production, and decrease mortality [3, 4]. In the last decade, multiple studies have reported that AGPs use has been associated with the rapid emergence of antibiotic-resistant microorganisms and high antibiotic residue levels in the consumable poultry products [5–10]. It has been suggested that the majority of antibiotic-resistant *E*. *coli* strains circulating in the community have been acquired from food animals, mainly from poultry [11]. The magnitude of these reports has raised numerous concerns and led to AGP restricting or banning action by several governments [12].

There is ample evidence of the chicken’s intestinal microbiota role in suppressing pathogen colonization through the modulation of the intestinal mucosa environment and the synthesis of antimicrobial compounds such as bacteriocins [13], short-chain fatty acids (SCFA) and hydrogen peroxide (H_2_O_2_) [14]. For instance, Enterobacteriaceae and other acid-sensitive pathogens are inhibited by SCFA production in the cecum, affecting the pH levels of the intestine and promoting dissipation of the proton motive force across the bacterial cell membrane [15]. Numerous studies show that the addition of dietary supplements such as probiotics, organic acids, and phytobiotics can positively modulate gut microbiota [16].

One of the promising alternatives to AGPs are probiotics (direct-fed microbes). The administration of probiotics in animal feed was reported to control intestinal pathogens [17, 18], enhance the intestinal health, and improve performance [19]. The potency of probiotics is linked to a strain and dose-specific relationship. In poultry production, the most frequently used bacterial probiotics include species of genera such as *Bacillus*, *Bifidobacterium*, *Enterococcus*, *Escherichia*, *Lactobacillus*, *Lactococcus*, *Streptococcus*, and a combination of undefined cultures [14, 20–22].

As with many application approaches, the mode of action of probiotics is not always clear. The mechanisms of probiotic action include competition with enteric pathogens for attachment sites, mucin secretion induction, immune modulation, cross-feeding, stabilization of the epithelial barrier, and the production of some inhibitory substances such as short-chain fatty acids, hydrogen peroxides and bacteriocins [16, 23, 24]. Studies on *Bacillus subtilis* PB6 probiotic show promising results. *Bacillus subtilis* PB6 significantly improves the intestinal morphology, growth performance, carcass traits, and controlled Necrotic Enteritis (NE) in broilers [19, 25]. However, the interactions of *B*. *subtillus* and *C*. *perfringens* with intestinal microbiota and gut health remain unexplored. NE is considered as one of the most devastating poultry diseases. An outbreak of NE in a flock may have a mortality rate as high as 50% [26]. The burden of NE in poultry is on the rise again after the forced exclusion of AGPs from the poultry feed and industry is now urging the scientific community to look for organic alternatives.

To date, most of the studies have focused on the immediate effect of NE challenge. The birds in industrial production often get NE approximately mid-production around day 18–20; however, this can be highly unpredictable. Studies that focus on controlling the clinical effects of NE closely post-challenge while the birds are showing clinical symptoms. Despite the high NE mortality rate, surviving birds recover relatively quickly and continue to grow to the end of the production cycle [27–28]. The effects continual probiotic supplementation has on the flock are cumulative and include post-NE recovery. There were studies investigating the influence of variables across the whole production cycle, including post-NE recovery, to observe and report long term adverse effects of NE challenge on performance measures [27–28].

The current study aims to present novel insights on the effectiveness of continual supplementation of a commercial AGP alternative *B*. *subtilis* probiotic on birds that suffered NE during production. We also used culture-free sequencing-based microbiota analysis combined with histomorphometric measurements and SCFA analysis to observe the long term effects of *B*. *subtilis* administration in NE challenged broiler model.

## Materials and methods

### Chicken management and housing

A total of 100 day-old broiler chicks (Ross 308) of mixed-sex were used in this trial. The chicks were randomly allocated into four dietary treatments and raised in a cage system; each treatment was further divided into five replicates with five birds per cage with a total of 25 birds per treatment. The experiment was performed for 40 days, with standard starter (0–21) and finisher diet (21–40 days). Broilers were raised in temperature and light controlled room under similar managerial and hygienic conditions. Birds had *ad libitum* access to water and feed and were maintained on a 24 h light schedule.

### Dietary treatments

Standard starter and finisher diets with isocaloric and isonitrogenous contents were offered in mashed form. Corn and soybean meal diet (corn-SBM) was formulated as recommended by the strain recommendation to meet or exceed recommendations in commercial practice in Saudi Arabia (S1 Table). All additives were supplemented by top dressing and were not included in the nutrient matrix. *Bacillus subtilis* PB6 probiotic was sourced by using commercial product (CloStat, Kemin Industries). Upon arrival, chicks were randomly distributed to one of the four treatments with 25 birds/treatment as follows: Treatment 1 (T1): negative control (No challenge, no *B*. *subtilis*); Treatment 2 (T2): positive control (*C*. *perfringens* challenge); Treatment 3 (T3): probiotic (*B*. *subtilis*, 0.5 g/kg); Treatment 4 (T4) probiotic and challenge: *B*. *subtilis* (0.5 g/kg) + (*C*. *perfringens* challenge). Necrotic enteritis was induced in broiler birds by a series of inoculation as described earlier [29] with minor modifications. Briefly, on the day 23, birds from NE challenged Treatments 2 and 4, were orally gavaged with *Eimeria* sp. using the Coccidia^™^-D vaccine at 13 times the recommended dose per bird, followed by *C*. *perfringens* type A strain inoculation at the rate of 4× 10^8^ CFU for three days on day 26, 27, and 28. The success of the NE model in challenged groups was confirmed through the necropsies of all infected birds starting from the day of *C*. *perfingens* inoculation onwards, carried out to confirm mortality cause. Characteristic signs of confluent necrosis and sloughing epithelium of the intestinal tract were recognized as caused by NE.

### Performance measurements

Broilers feed intake (FI) was calculated weekly by subtracting the amount of feed rejected from the total offered feed. Average body weight gain (BWG) was measured weekly, and average feed conversion ratios (FCR) were adjusted for mortality and computed for each experimental unit. Production efficiency factor (PEF) was determined by using the formula:

$$PEF=\frac{Livability\times Live\;weight(kg)}{Age\;in\;days\times FCR}\times 100$$

### Sample processing

At the end of the trial (day 40), cecum contents were collected aseptically, 14 birds from each of the different treatments were chosen randomly for microbiota and SCFA analysis. Samples were immediately placed on ice, transferred to the lab and stored at -80°C. For histological analysis, tissue from six birds per treatment selected randomly from all pen replicates was removed aseptically from ileum mid-section, washed in phosphate-buffered saline (PBS) and fixed in 10% buffered formaldehyde.

### 16S rRNA-based analysis of broilers cecal microbiota composition

Total DNA for microbiota analysis was extracted using a protocol described by Stanley et al., [30] and Yu and Morrison [31] with minor modification by including additional column wash. Quantity and quality of DNA were measured using a Nanodrop spectrophotometer. The microbiota composition of broilers was determined by amplification and sequencing of 16S ribosomal RNA (rRNA) genes and was performed using specific primers targeting the V3–V4 hypervariable regions; forward ACTCCTACGGGAGGCAGCAG, and reverse GGACTACHVGGGTWTCTAAT. Primers contained barcodes, spacers and Illumina sequencing linkers applying the method that has been previously proposed by Fadrosh et al., [32]. Subsequently, the sequencing library was prepared following the manufacturer’s protocol (Illumina Inc., San Diego, CA, USA). Sequencing was performed on the Illumina MiSeq platform using 2x300 bp paired-end sequencing.

### Sequencing and statistical analysis

Sequencing reads generated by Illumina MiSeq were initially processed and analyzed using Quantitative Insights into Microbial Ecology (QIIME v.1.9.1) [33]. Paired-end joining Fastq-Join algorithm allowed no mismatches within the region of overlap. Sequences with Phred quality threshold greater than 20 were retained in the analysis. The assembled sequences were clustered into operational taxonomic units OTUs at 97% similarity using UCLUST algorithm [34] and investigated for chimeric sequences using Pintail [35]. All taxonomic assignments were done in QIIME against the GreenGenes reference OTU database and QIIME default arguments [36]. Weighted and unweighted Unifrac matrixes were calculated in QIIME, with 99,999 permutations and OTUs with a relative abundance of lower than 0.01% were filtered out. After quality filtering, 52 cecum samples were successfully sequenced. The data analysis was performed using Hellinger transformed rarefied data [37], square-root transformed, and TSS normalized. Further data exhibitions and visualization were done using Calypso (http://cgenome.net/calypso/) [38]. Alpha diversity was evaluated using Chao1, Shannon, Richness, Evenness, and Simpson indexes. The figures comparing relative abundance show untransformed data. 2-way PERMNAOVA (Primer 7e) was used to inspect the influence of the two factors (NE challenge and *B*. *subtilis*) and their interactions. ANOVA was used to detect the significance of the differences between the groups. Multivariate data visualization and multivariate statistical testing were examined by implementing the supervised multivariate redundancy analysis (RDA) using 999 permutations. The complete sequencing dataset is publicly available on the MG-RAST server (http://metagenomics.anl.gov/) with library ID mgl758080.

### Short-chain fatty acid extraction and analysis

SCFAs were extracted from the cecal contents using an acidified water-extraction method described by Zhao et al., [39] with some modifications. Briefly, 0.2 g of cecal content was suspended in at 5 mL of water and homogenized by vortexing. Subsequently, the pH of the suspension was adjusted to 2–3 by adding 5M hydrochloric acid (HCl), and then 1 mL of Acetonitrile chromatography grade (Sigma–Aldrich) to enhance the perception of other contaminants. Samples then were kept at room temperature for 10 min with occasional shaking, followed by centrifuging for 20 min at 5000 rpm, resulting in a clear supernatant. One mL of the final extract was filtered using a 0.2 μm cellulose regenerated PTFE syringe filter and transferred to glass auto-sampler vials. Filtered samples were evaporated to dryness at 70 °C for 48 hours, then re-dissolved in acetonitrile, vortexed for 1 min and the volume of the extract was adjusted to 50 μl by a gentle stream of high purity N2 gas before injection into Agilent 7890A GC system gas chromatography-5975 C inert MSD model mass spectrometer (GC–MS) (Agilent Technologies, Palo Alto, CA). Acetic acid (C2) (99.9%), Propionic acid (C3) (99.9%), Butyric acid (C4) (99.5%), *i*-butyric acid (*i*-C4) (99.0%), n-valeric acid (C5) (99.3%) and *i-*valeric acid (*i*-C5) (99.0%) were purchased from Dr. Ehrenstorfer (Augsburg, Germany). All standards concentrations were adjusted to 1000 ug/ml (ppm) in methanol. GC-MS on an Agilent (Palo Alto, CA) 7890A was used and operated in total ion chromatogram (TIC) scan mode to determine the retention time of each SCFA compound and the mixture of SCFAs. The GC-MS was equipped with an Agilent350 °C column (30 m x 250 μm x 0.25 μm). Separation of the SCFAs was done as previously described [40–43]. GC/MS was operated in a single ion monitoring (SIM) mode according to the following instrumental parameters. The oven program was 50 °C for 1 min, then 6 °C/min to 100 °C for 1 min, then 25 °C/min to 270 °C for 1 min, (a total of 18.133 min run program), and 2 min (Post Run) 300 °C. Sample volume of 2 μL was injected at heater on 250 °C using helium as a carrier gas in a split-less mode. The pressure was maintained at 11.747 psi at 24.4 mL/min total flow. Calibration curves were constructed using standard stock solutions that were prepared from a stock concentration of 1000 ppm for acetic acid, propionic acid, butyric acid, *i*-butyric acid, n-valeric acid and *i*-valeric acid.

### Histological analysis

Two-centimetre tissue samples were collected from the ileum (mid-section), fixed in 10% (vol/vol) buffered formaldehyde for approximately 72 h, followed by dehydration in graded alcohol, and embedded in paraffin. The 5 μm sections were cut using a microtome and stained with hematoxylin and eosin (H&E) stain following Samanya and Yamauchi [44]. Histological images were scanned using a Nikon Eclipse Ni-U microscope with a camera (Nikon, Tokyo, Japan) at magnifications of (4x, 10x, 40x). Histomorphometric indices included villi height, villi width, and total villi area was estimated based on a minimum of 10 well-oriented longitudinal villi were assessed per intestinal section of the small intestine per bird using an IX71 Inverted Olympus Microscope (eyepiece: WH10x; objective lens: 4x), digitalized using image (Olympus DP72 microscope digital camera (Olympus NV, Aartselaar, Belgium) and analyzed using CellSens digital imaging software for research application) tools. Morphometric analyses were performed from six individual birds as replicates per treatment group. Villus height and width data were used to calculate villus surface area using the formula:

VillusSurfaceArea:[2π×(villuswidth/2)×villuslength]

### Macroscopic intestinal lesion scores

Inspection of broilers carcasses for necrotic enteritis (NE) lesions in the duodenum, ileum, and jejunal regions of the intestine was performed using the grading method described by Gholamiandehkordi et al., [45]. The lesions were graded from 0 to 3 based on their severity. Grade 0 = clear intestine with no lesions, 1 = focal necrosis with ulceration, 2 = patches of necrosis from 2 to 3 centimeters’ long, and 3 = diffuse necrosis representative of field cases.

Collected data was evaluated using ANOVA for a complete randomized block design, using the general linear models (GLM) procedure of SAS software (SAS, 2009). Least significant difference (LSD) test was applied to compare the treatment groups means when the treatment effect was significant at *P*-value <0.05. Both histological analysis and lesion grading were done by the same histopathology expert who was blinded to the treatments and had only bird number ID associated with the slide or tissue sample.

### Ethics statement

The study was approved by the Research Ethics Committee, Deanship of Scientific Research, Vice-Rectorate for Graduate Studies & Scientific Research at King Saud University project approval number KSU-SE-18-38. All methods were performed in accordance with the Gloucestershire County Council’s (GCC) Animal Welfare Act endorsed by Saudi Arabia and were approved in Royal Decree No. (M / 44). The birds were euthanized by quick decapitation away from the other birds.

## Results

### Effect of dietary treatments on growth performance

Mortality was present only in NE challenged treatments, T2: positive control (NE challenge) with 9 mortalities and T4 (*B*. *subtilis* probiotic and NE challenge) with 4 mortalities. Neither T1 (no NE, no *B*. *subtilis*) nor T3 (*B*. *subtilis* only) had any mortalities. Broiler’s growth performance was calculated and analyzed following different stages of the trial; pre-infection (0-21d), NE (21-28d), post-infection (28-35d), and cumulative performance (0-35d) (Table 1). During the pre-infection period, there was no significant difference between all groups (T1-T4) in all measured parameters. Significant variation was observed in NE period where birds belonging to T2, T4, and T3 had the highest FCR values respectively (1.55.6, 1.542, 1.473) (*P =* 0.003); however, probiotic supplementation did not significantly improve FCR in challenged birds as no significance between T2 and T4 was seen. Following the post-infection period, FCR exhibited highly significant variations (*P =* 0.0001), with negative control (T1) and *B*. *subtilis* PB6 based-probiotic (T3) showing the lowest FCRs. Birds in NE challenge (T2) had the highest FCR value. Noteworthy, birds that underwent NE challenge and had their diet supplemented with *B*. *subtilis* PB6 (T4) showed significantly lower FCR compared to NE challenged without probiotic supplementation. This difference was observed only in the post-NE period. Overall, the highest final body weight values were observed in *B*. *subtilis* PB6 based-probiotic (T3), followed by T1, while the lowest values were scored in T2 and T4 (*P =* 0.042).

**Table 1:** Effect of *B*. *subtilis* PB6 based-probiotic supplementation and *C*. *perfringens* challenge on cumulative feed intake, body weight gain, feed conversion ratio and production efficiency factor of broilers during different stages of the experiment.

Treatment group	Performance																	
	d 0–21 (Pre-Infection)				d 21–28 (Necrotic Enteritis)				d 28–35 (post-NE)				d 0–35 (Overall)					Livability %
	FI, g	BWG, g	FCR, g:g	PEF	FI, g	BWG, g	FCR, g:g	PEF	FI, g	BWG, g	FCR, g:g	PEF	FI, g	BWG, g	FCR, g:g	PEF	Final body weight, kg	
**T1**	971.7	804.3	1.209	333.79	738.9^a^^b^	540.8^a^	1.367^b^	362.6^a^	858.5	587.8	1.461^c^	378.6^a^^b^	2569.1	1932.9	1.329^b^	415.8^a^^b^	1.934^a^^b^	100^a^
**T2**	970.9	795.3	1.221	325.9	669.6^b^	430.9^b^	1.556^a^	291.1^b^	914.5	546.9	1.674^a^	284.4^c^	2555.0	1773.0	1.441^a^	329.7^b^	1.800^b^	93^b^
**T3**	927.1	764.8	1.209	327.7	753.4^a^	512.3^a^^b^	1.473^a^	326.5^a^^b^	892.4	597.9	1.493^c^	385.9^a^	2572.9	1875.1	1.372^b^	419.3^a^	2.009^a^	100^a^
**T4**	976.2	788.9	1.238	319.2	666.5^b^	433.4^b^	1.542^a^	393.3^b^	915.1	582.6	1.570^b^	322.3^b^^c^	2557.8	1804.9	1.417^a^	334.2^a^^b^	1.845^b^	97^a^^b^
**SEM±**	40.3	30.7	0.015	8.1	24.5	19.6	0.027	11.52	28.8	18.4	0.023	17.8	63.2	43.4	0.013	27.2	0.048	1.172
***p-value***	NS	NS	NS	NS	.056^NS^	0.005**	0.003**	0.006**	NS	NS	0.0001***	0.0048	NS	NS	0.0008	0.06^NS^	0.042*	0.0018**

T1: Negative Control; T2: Positive Control, NE challenge; T3: *B*. *subtilis* PB6 probiotic, unchallenged; T4: *B*. *subtilis* PB6 probiotic, NE challenge; BWG, bodyweight gain; FI, feed intake; FCR, feed conversion ratio; PEF, production efficiency factor.

^abcd^Means in the column with different superscripts differ significantly. NS, not significant.

### Effect of dietary additives on ileum histomorphological measurements

**Fig 1:**
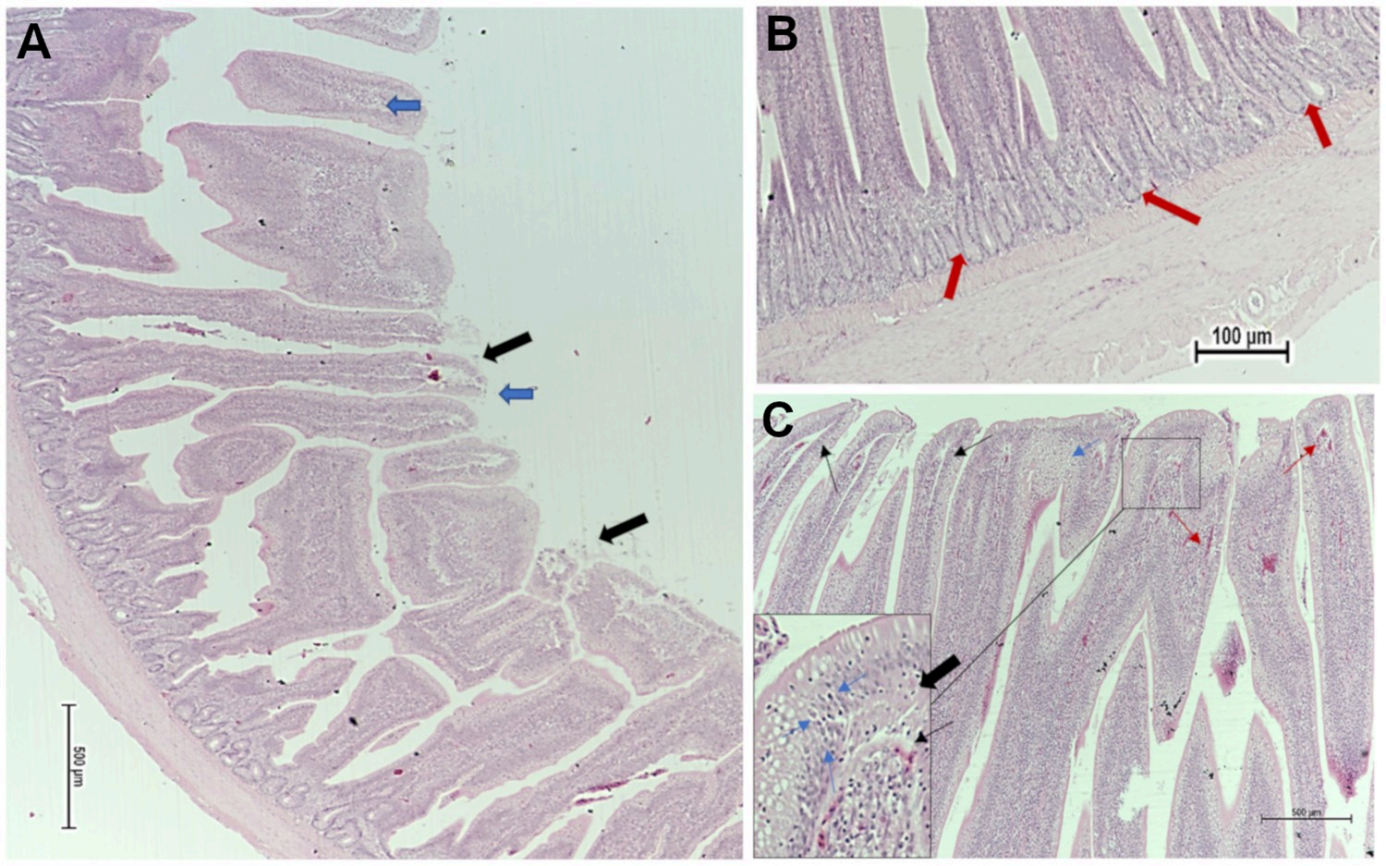
Ileum photomicrograph of NE challenge (T2), (A) illustrating disorganized villi with fusion and flattening (blue arrows), and (B) crypt hyperplasia (red arrows). (C) Edema spreading throughout the structure of the lamina propria with the edematous spaces clearly defined by deposition of light pink stained material (black arrows), mild infiltration of lymphocytes and polymorphonuclear cells (heterophil influxes) (thin red arrows). Noteworthy is a marked separation of the epithelial cells from the basement membrane (thin black arrows). Several cells in the lamina propria show signs of necrotic cell death, as well as enterocytes, show more advanced signs of necrosis such as cytoplasmic vacuolization, karyorrhexis, nuclear pyknosis, and goblet cells metaplasia (thin blue arrows). Haematoxylin and Eosin (H&E) stain.

**Fig 2:**
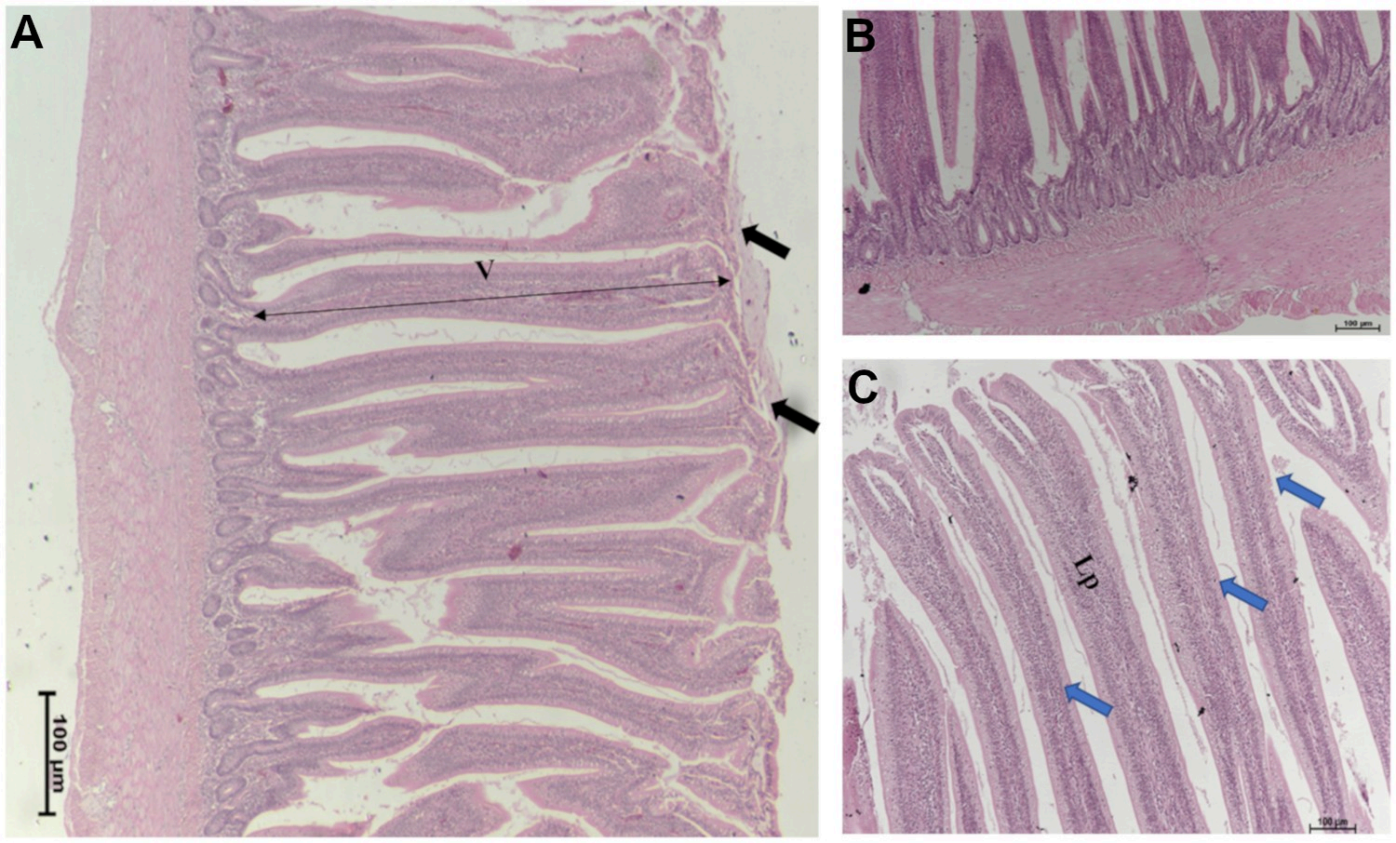
Ileum photomicrograph of broilers under NE challenge and *B*. *subtilis* PB6 probiotic supplementation (T4), (A) Illustrating some separation and presence of necrosis coagulation on villus superficial tips (black arrow). (B) and (C) Less inflammation and edema observed through the structure of the lamina propria, Broad and thickened villus tips showing relatively tall organized intestinal villi (blue arrows).

The impact of different nutritional additives on ileum histomorphometric measurements, including villus length, width, and surface area in broiler chickens at (40 d) are presented in S2 Table. Positive control group T2 (*C*. *perfringens* challenge) represented the narrowest villi, wherein, the largest average villi width (88.8 μm), was observed in T3 (*B*. *subtilis* PB6 based-probiotic; *B*. *subtilis*) (*P* = 0.052). Figs 1 and 2 represent the histopathological changes observed in the ileum from positive control group T2 (NE challenged) and T4, *B*. *subtilis* supplemented /NE challenged group. Birds that were challenged with NE (T2) exhibited multiple mild microscopic presentations of necrotic enteritis starting with disorganized villi with fusion and flattening, infiltration of lymphocytes and polymorphonuclear cells, crypt hyperplasia, edema, separation of the epithelial cells from the basement membrane, various cells showing necrotic cell death signs such as cytoplasmic vacuolization, karyorrhexis, nuclear pyknosis, and goblet cells metaplasia (Fig 1). However, broilers under NE challenge and probiotic supplementation (*B*. *subtilis*) (T4) represented broad and thickened villus tips showing relatively tall organized intestinal villi and necrosis coagulation present on superficial villus tips, with milder inflammation and edema (Fig 2).

### Effect of dietary supplements on intestinal lesion scores

The result of lesion scores in duodenum, jejunum, and ileum regions at 40 days is presented in Table 2. Highly significant variations were observed across duodenum, jejunum, and ileum sections, between T2 (NE challenged) and T4 (NE challenged and supplemented with *B*. *subtilis*) where T4 group had significantly lower intestinal lesions scores compared to T2 in duodenum, jejunum and ileum (Table 2). There were no significant differences in lesion scores in other group comparisons.

**Table 2:** Effect of *B*. *subtilis* supplementation and *C*. *perfringens* challenge on intestinal macroscopic necrotic enteritis lesion scores (0–3) of broilers at 40 d.

Treatment group	Macroscopic NE Lesion Scores (points)		
	Duodenum	Jejunum	Ileum
T 2: Positive Control, *C*. *perfringens* Challenge	1.86^a^	1.21^a^	1.07^a^
T 4: *B*. *subtilis*, *C*. *perfringens* Challenge	0.86^b^	0.40^b^	0.50^b^
**SEM**^1^	**±**0.188	**±**0.158	±0.108
***p-Value***	0.0001***	0.0001***	0.0001***

^1^ SEM: standard error of the mean ^ab^ Means values within a column with different superscripts are significantly different *, *P* <0.05; **, *P* <0.01; ***, *P* <0.001, NS, not significant.

### Overview of microbiota structure

Overall microbiota of birds used in this trial was characterized by the dominance of five major phyla, where Firmicutes was the most dominant, followed by a high number of sequences assigned to Bacteroidetes, in addition to Actinobacteria, Tenericutes, and Proteobacteria (S1 Fig). Among classified culturable genera, cecal microbiota profile was composed mostly of high abundance of *Faecalibacterium* (approximately 40% of total reads), and *Ruminococcus* followed by *Oscillospira*, *Coprococcus*, *Bacteroides*, *Lactobacillus*, *Blautia*, *Eubacterium*, *Dorea*, *Coprobacillus*, and *Eggerthella*. The remaining identified microbes were unclassified and unculturable genera, as shown in S1 Fig.

### Influence of probiotic supplementation and NE challenge on broilers cecal microbiota composition

We performed 2-way PERMANOVA analysis with 99999 permutations at a phylum, genus and OTU level. The tables with a direct screen capture of Primer 7e outputs that include details of both analysis and results are given in S3A–S3C Table at a phylum, genus and an OTU level. At a phylum level, NE challenge (*P =* 0.011) and interaction between probiotic supplementation and NE challenge (*P =* 0.034) were significant while the effects of *B*. *subtilis* probiotic additive were not (*P =* 0.42). At the genus level, there were no significant differences; however, NE challenge was marginally affected (*P =* 0.064). At an OTU (most comparable to species) level, NE challenge (*P =* 0.0079) and interaction between probiotic supplementation and NE challenge (*P =* 0.032) were significant while the effects of *B*. *subtilis* probiotic additive were marginal (*P =* 0.065).

**Fig 3:**
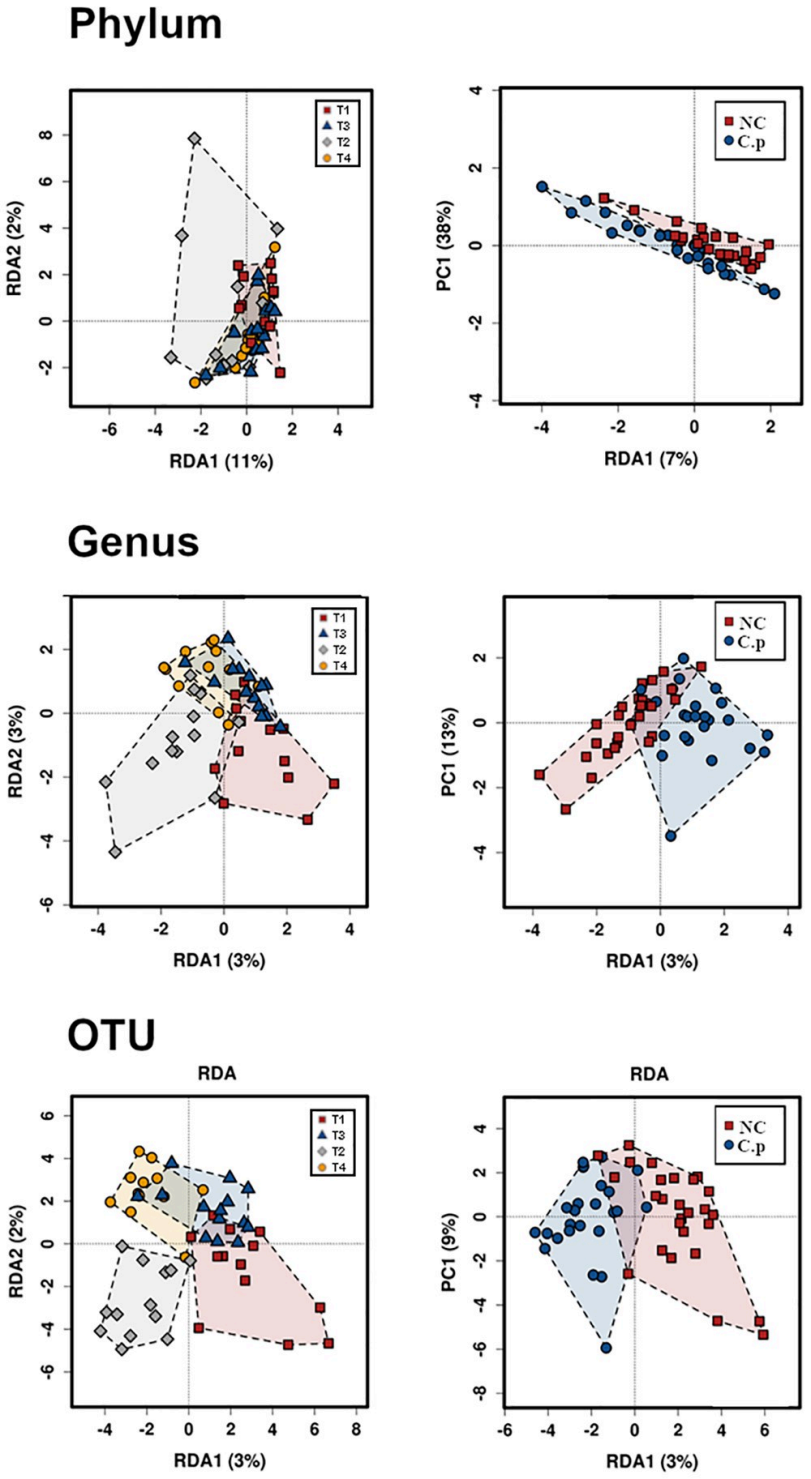
The treatment differences in cecal microbiota composition. Multivariate redundancy analysis RDA plots at a phylum (*P* = 0.002), genus (*P =* 0.039), and OTU (*P* = 0.029) levels showing all 4 groups (left). RDA plots showing the effect of NE challenge on broilers beta diversity at a phylum (*P* = 0.019), genus (*P =* 0.02), and OTU (*P* = 0.011) levels (right). C. p: *C*. *perfringens;* CT: control groups.

In addition to 2-way PERMANOVA that allows for an investigation of factors interaction, we performed other multivariate analysis such as RDA that allows visualizing of the group to group similarity as well as providing a measure of the significance of each factor but not their interactions. RDA illustrated significant variations in microbiota composition at a phylum level between the four selected treatment groups (*P =* 0.002) with no significant influence of probiotic on the microbiota composition (*P =* 0.227). However, a significant difference (*P =* 0.019) was observed between NE challenged (T2 and T4) and NE unchallenged birds (T1 and T3). At the genus level, the RDA analysis showed significant variability in the microbiota between the 4 groups (*P =* 0.039) and NE challenge effect (*P =* 0.02). Finally, at an OTU level, multivariate RDA analysis indicated a significant variation between the treatment groups (*P =* 0.029) and in the NE challenge (*P =* 0.011). As in 2-way PERMANOVA, there were no significant differences in microbiota due to the presence of a probiotic supplement at any of the taxonomic levels (Fig 3).

Comparing the alpha diversity showed that no significant differences were observed between the 4 treatments inspecting various diversity matrices such as Shannon, Richness, Chao1, Evenness, and Simpson indexes. Moreover, when we separated the birds into two categories based on whether they received *B*. *subtilis* probiotic as a dietary supplement (T3 and T4) or not (T1 and T2), we also observed no significant differences. However, when we investigated the influence caused by NE challenge on diversity (T1 and T3), we found that broilers inoculated with the pathogen had significantly lower Richness (*P* = 0.047) while it did not affect other parameters (S2 Fig).

**Fig 4:**
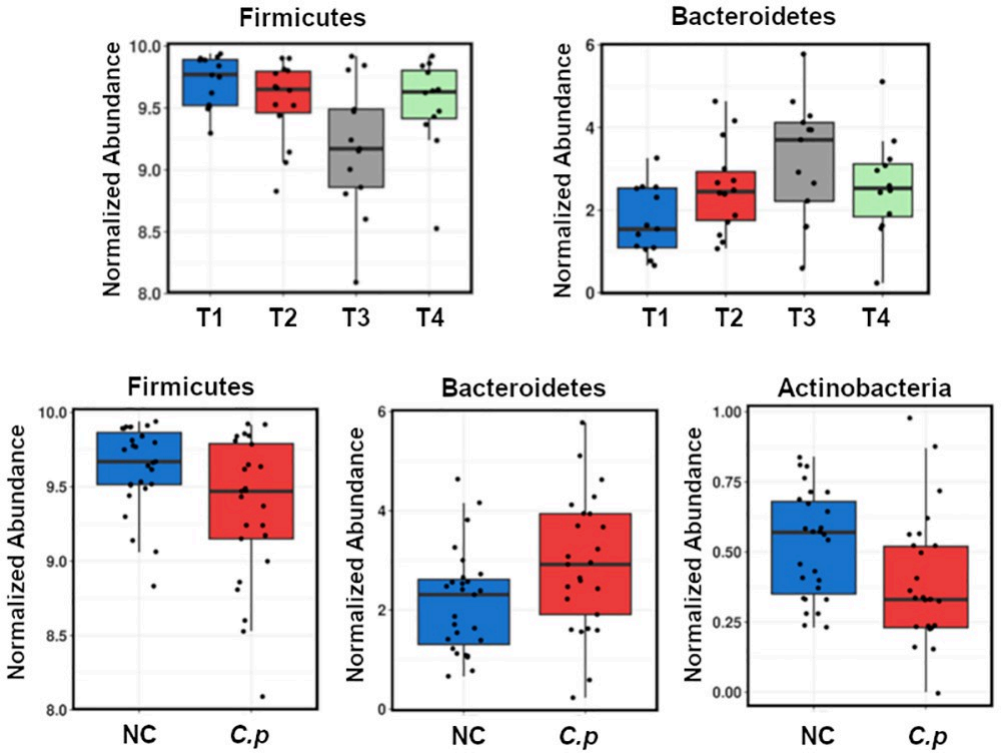
Most significant univariate alterations observed at the phylum level at the end of the production cycle in birds recovered from NE. C. p: *C*. *perfringens*; NC: non-challenged. Top four-bar boxes represent ungrouped data.

We then proceeded to investigate the individual influence of taxa at these different taxonomic levels (phylum, genus, and OTU) using one-way ANOVA test between the four treatment groups, illustrating that major differences were caused by changes in Firmicutes and Bacteroidetes phyla (P = 0.0075 and 0.02, respectively). There was an increase in Bacteroidetes at the expense of Firmicutes, and marginally decreased Actinobacteria in challenged groups (Fig 4). Comparing probiotic administered groups (T3 and T4) to non-supplemented groups (T1 and T2) revealed a significant decrease in the phylum Proteobacteria (P = 0.019).

**Fig 5:**
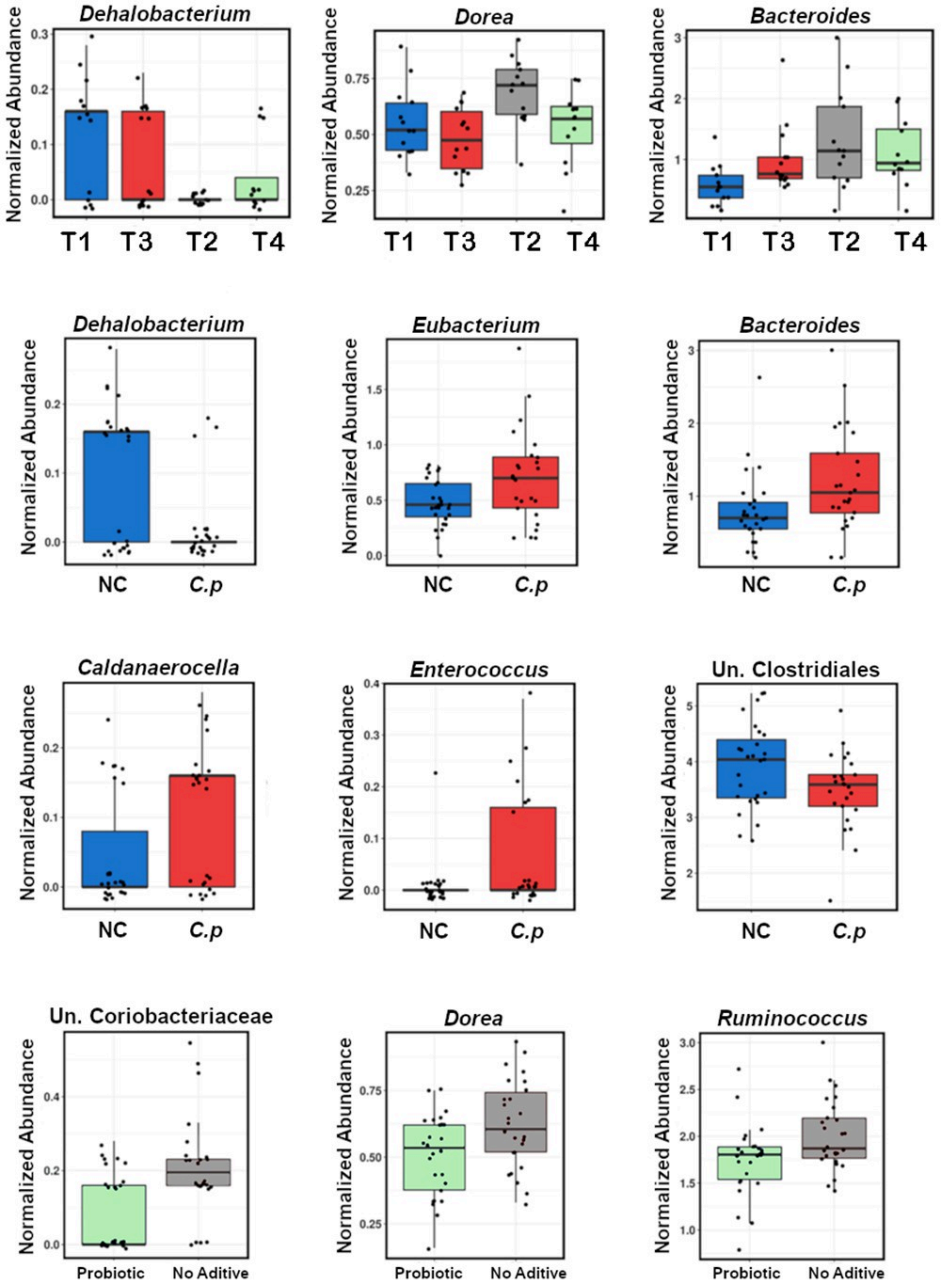
Genera showing significant changes between the selected treatment set and in correlations with the presence of NE challenge or probiotic administration, *C*.*p*: *C*. *perfringens*; NC: Non-challenged. Top four-bar boxes represent ungrouped data.

At the genus level, the abundance of *Dehalobacterium* was significantly higher in control group T1 (*P* = 0.0028) while *Dorea*, *Bacteroides*, *Eubacterium*, *Caldanaerocella*, and *Enterococcus* were increased in challenged birds (T2 and T4). Probiotic supplemented groups showed a decrease in *Dorea*, *Ruminococcus*, and several unclassified genera (T1 and T3) (Fig 5). Linear discriminant analysis and effect size method (LEfSe) singled out the genera that are most likely to describe the differences between groups (S3 Fig).

### Dietary supplementation and bacterial challenge influence on short-chain fatty acid production

**Fig 6:**
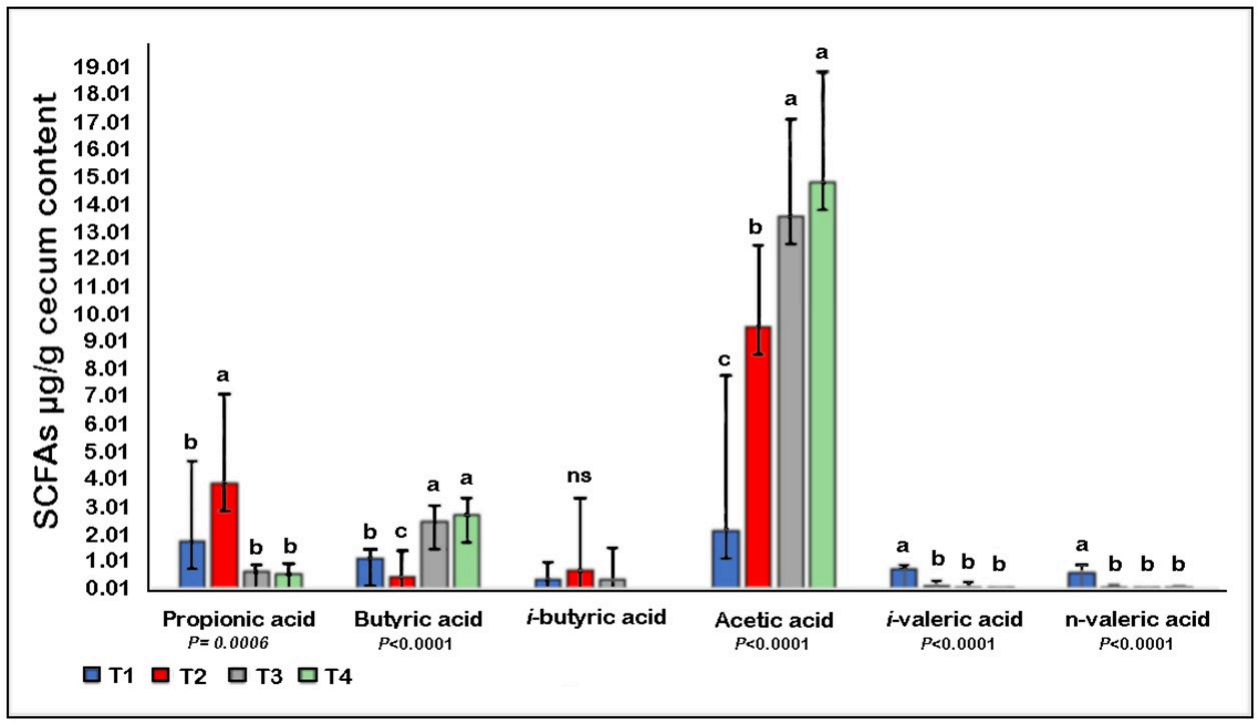
Effect of NE challenge and *B*. *subtili*s PB6 based-probiotic supplementation on six SCFA concentrations in broiler chicken cecum. ^abc^ Means in the bar with different superscripts differ significantly. ns: not significant. Error bars represent standard deviation (SD).

There was a significant increase in cecum propionic acid with *C*. *perfringens* challenge (T2). Both of the *B*. *subtilis* supplemented groups (T1 and T3) were characterized by a significant rise in acetic acid concentration, while n-valeric acid was significantly higher in the control group (T1). Further, *B*. *subtilis* administration was associated with a significant increase in butyric acid (*P*<0.0001) (Fig 6).

## Discussion

We performed an in-depth investigation of a promising AGP alternative, *B*. *subtilis* commercial probiotic. *B*. *subtilis* supplementation demonstrated its capacity in improving bird’s performance in the recovery stage following NE challenge. Dietary probiotics have previously shown an association with increased broilers productivity, including improved body weight gain, better FCR, improved the intestinal morphological status, and decreased mortality [27, 46, 47]. The inhibitory capacity of *B*. *subtilis* PB6 against some enteric pathogens invasion in chicken is well documented [48]. Our results are in agreement with Jayaraman et al., [49] who reported that *B*. *subtilis* PB6 supplementation reduced FCR and improved body weight, especially in broilers challenged with *C*. *perfringens*. The supplementation with *B*. *subtilis* PB6 reduced NE intestinal lesion scores, which might be attributed to the production of previously reported anti-clostridial factors produced by *B*. *subtilis* PB6 [48] and improved post-NE recovery. Additionally, according to the data presented in Table 1, FCR was not different between challenged (T2) and challenged with probiotic supplemented (T4) groups before or during NE challenge, nor using total cumulative trial data. The significant difference existed only during post-NE recovery period where probiotic supplemented challenged group had significantly better FCR.

High richness estimates are associated with good health and indicate the availability of a niche and nutrient resources for maintaining a large number of species. Wherein, decreased richness and diversity of intestinal microbiota is considered as a risk factor for dysbiosis and several gastrointestinal complications [50]. In the current trial, we observed a significant decline in richness in birds under NE challenge, reflecting the negative effects on bird’s health [51]. This reduction in richness was not ameliorated with *B*. *subtilis* substitution.

Both 2-way PERMANOVA and an RDA analysis agreed that *B*. *subtilis* supplementation does not affect microbiota per se; however, significant PERMANOVA interactions indicate that *B*. *subtilis* supplementation does have an influence on NE challenge induced changes in the microbiota which are substantial at all taxonomic levels. Gram-positive Firmicutes phylum harbors many health-promoting bacterial groups such as *Lactobacillus* and is recognized as a primary pool of probiotic species [52] while also being a host of some pathogens, including *C*. *perfringens*. Some members of gram-negative Bacteroidetes phylum are known for their ability to degrade high molecular weight compounds such as carbohydrates and proteins, thereby supporting the host in acquiring higher nutrients [53]. One of the major shifts observed in the infected bird’s microbiota was a significant increase of Bacteroidetes and reduction of Firmicutes, however, as both these phyla are predominantly beneficial commensals, the major effect on this shift is inconclusive. *B*. *subtilis* supplementation significantly reduced pathogen-harboring phylum of Proteobacteria, which may have a major significance. The abundance of evidence that expansion of Proteobacteria highly compromises the ability of the host to maintain intestinal and overall health was explored to detail previously [54]. The study suggested that increase in Proteobacteria should be used as a marker of dysbiosis, a pre-disease state of microbiota imbalance or maladaptation characterized by the lack of ability of the microbiota to control pathogen overgrowth and waste product accumulation. Our data demonstrate the ability of *B*. *subtilis* to ease dysbiosis, which might be an additional benefit in a range of challenging intestinal situations.

We observed a sharp decline in the abundance of *Dehalobacterium*, a novel, and underexplored genus, during the *C*. *perfringens* challenge. *Dehalobacterium* decrease was suggested as a biological marker in patients at high risk of developing bloodstream infections in humans undergoing chemotherapy [55]. However, the casual connection of *Dehalobacterium* reduction and the role it may play in the onset of NE warrants further investigation.

Chicken feed is mainly grain-based and fibrous, and cannot be completely metabolized by the host. It has been reported that 20% of poultry intestinal microbiota functional genes were associated with carbohydrate metabolism [56], resulting in the production of multiple SCFAs in the chicken gut, primarily acetate, propionate, and butyrate [57]. Thus, SCFAs levels might be used as an indication of health-related bacterial groups and a contributor to enhanced chicken growth performance [58]. *B*. *subtilis* administration was found to be associated with a significant increase in acetic acid and butyric acid concentrations (*P<*0.0001). Butyric acid has been reported in several studies to be of considerable benefit in the bird’s energy status, growth performance, and villus development [59, 60]. These reports support the data obtained in this study where we observed an improvement in post-NE performance and increased surface area of intestinal villus of probiotic-supplemented birds. Probiotic supplementation increased the concentration of butyric acid in both challenged and not challenged supplemented groups. Interestingly, Timbermont et al., [61] reported that supplementing chicken feed with butyric acid contributed to the prevention of NE in NE challenged broilers, which could contribute to the *B*. *subtilis* protective effect.

In conclusion, based on intestinal condition in NE recovered birds, our results demonstrate that *B*. *subtilis* supplementation did assist the birds in dealing with NE outbreak long term recovery. *B*. *subtilis* supplementation improved SCFA profile without causing major disturbance to microbiota. However, subtle changes to microbiota profile introduced by probiotic supplement were beneficial with reduction of the major pathogen, disease and dysbiosis related phylum Proteobacteria and with significant interaction and influence on the effects of NE challenge on intestinal communities based on PERMANOVA interaction significance. More research is suggested to further investigate the mechanisms involved.

## Supporting information

S1 Table Ingredients and calculated nutrient analysis of broilers starter and finisher diets. (PDF)

S2 Table Effect of different dietary supplementation and bacterial challenges on ileum histomorphometric measurements of broilers at (40 d). (PDF)

S3 Table Results from Primer 7e 2-way PEMANOVA analysis at a phylum (A), genus (B) and an OTU level (C). (PDF)

S1 Fig Cecal microbiota composition of broilers within different groups; (A) the most dominant phyla, and (B) the 20 most abundant genera. (PDF)

S2 Fig Impact of challenge on *C*. *perfringens* impact on Alpha diversity, lower Richness in association with challenge (P = 0.047) (left) marginally influenced Chao1 index (P = 0.089) (right), NC: Non-challenged. (PDF)

S3 Fig Linear discriminant analysis and effect size method (LEfSe) showing the genera that are most likely to describe the differences between groups. (PDF)
