# Supplementary material for: Bacillus subtilis PB6 based probiotic supplementation plays a role in the recovery after the necrotic enteritis challenge
Source: PLoS One. 2020 Jun 18;15(6):e0232781. doi: 10.1371/journal.pone.0232781 (PMC7302482; doi:10.1371/journal.pone.0232781)
Supplement: S1 Table — (PDF) [file pone.0232781.s001.pdf]

Table S1. Ingredients and calculated nutrient analysis of broilers starter and finisher diets

**Ingredients**	**Starter**	**Finisher**
Yellow corn	50.635	65.39
Soybean meal	42.40	20.70
Wheat bran	0.00	0.60
Corn Gluten meal	0.00	0.70
Choline chloride CL 60	0.05	0.05
Corn oil	3.60	3.00
Dicalcuim Phosphate DCP	1.270	1.027
Ground Limestone	1.080	1.04
Salt	0.300	0.30
Phytase xp 10000 TPT	0.005	0.005
DL-methionine	0.295	0.22
Lysine-HCL	0.080	0.36
Threonine	0.085	0.11
Vitamin- Mineral premix[1]	0.200	0.200
Total	100	100
Analysis		
ME, kcal/kg	3000	3200
Crude protein, %	23.0	19.5
Non phytate P, %	0.48	0.359
Calcium, %	0.96	0.81
D. Lysine, %	1.28	1.03
Sulfur amino acids, %	0.85	0.8
Threonine, %	0.86	0.69

[1]Vitamin-mineral premix contains in the following per kg: vitamin A, 12000000 IU; vitamin D3, 5000000 IU; vitamin E, 80000 IU; vitamin K3, 3200 mg; vitamin B1, 3200 mg; vitamin B_2, 8600 mg; vitamin B_3, 65000 mg; pantothenic acid, 20000 mg; vitamin B_6, 4300 mg; biotin 220 mg; antioxidant (BHA+BHT), 50000 mg; B_9, 2200 mg; B_{12}, 17 mg; copper, 16000 mg; iodine, 1250 mg; iron, 20000 mg; manganese, 120000 mg; selenium, 300 mg, and zinc, 110000 mg.
