# Supplementary material for: Bacillus subtilis PB6 based probiotic supplementation plays a role in the recovery after the necrotic enteritis challenge
Source: PLoS One. 2020 Jun 18;15(6):e0232781. doi: 10.1371/journal.pone.0232781 (PMC7302482; doi:10.1371/journal.pone.0232781)
Supplement: S2 Table — (PDF) [file pone.0232781.s002.pdf]

Table S2. Effect of different dietary supplementation and bacterial challenges on ileum histomorphometric measurements of broilers at (40 d).

**Treatment group**	**Histomorphometric Measurements**		
	L (µm)	W (µm)	SA (µm)
Negative Control, basal diet, unchallenged	624.8	75.9[ab]	0.147
Positive Control, *C. perfringens* Challenge	566.3	75.6 [ab]	0.142
CloStat®**,** unchallenged	609.1	88.8[a]	0.171
CloStat®**,** *C. perfringens* Challenge	603.4	67.7[b]	0.130
**SEM**[1]	±20.9	±5.39	±0.011
p-Value	0.2431[NS]	0.052*	0.088 [NS]

L: villus length; W: villi width; SA: surface area,[1] SEM: standard error of the mean [abc] Means values within a column with different superscripts are significantly different *, P <0.05; **, P <0.01; ***, P <0.001, NS, not significant.
