# Supplementary material for: Bacillus subtilis PB6 based probiotic supplementation plays a role in the recovery after the necrotic enteritis challenge
Source: PLoS One. 2020 Jun 18;15(6):e0232781. doi: 10.1371/journal.pone.0232781 (PMC7302482; doi:10.1371/journal.pone.0232781)
Supplement: S3 Table — (PDF) [file pone.0232781.s003.pdf]

Table S3. Results from Primer 7e 2-way PEMANOVA analysis at a phylum (A), genus (B) and an OTU level (C)

Table 3A – Phylum level: abbreviations Factor 1- NE challenge (Cp) and Factor 2 -probiotic additive (Ad)

PERMANOVA
Permutational MANOVA

Resemblance worksheet
Name: Resem2
Data type: Similarity
Selection: All
Resemblance: S17 Bray-Curtis similarity

Sums of squares type: Type III (partial)
Fixed effects sum to zero for mixed terms
Permutation method: Permutation of residuals under a reduced model
Number of permutations: 99999

Factors

Name	Abbrev.	Type	Levels
Cp	Cp	Fixed	2
Aditive	Ad	Fixed	2

PERMANOVA table of results

Source	df	SS	MS	Pseudo-F	P(perm)	Unique perms	P(MC)
Cp	1	325.9	325.9	4.7139	0.0114	95736	0.0123
Ad	1	61.781	61.781	0.89361	0.4206	95735	0.4188
CpxAd	1	240.56	240.56	3.4795	0.0342	95740	0.0351
Res	48	3318.5	69.136				
Total	51	3951.5					

Details of the expected mean squares (EMS) for the model

Source	EMS
Cp	1*V(Res) + 25.923*S(Cp)
Ad	1*V(Res) + 25.923*S(Ad)
CpxAd	1*V(Res) + 12.961*S(CpxAd)
Res	1*V(Res)

Construction of Pseudo-F ratio(s) from mean squares

Source	Numerator	Denominator	Num.df	Den.df
Cp	1*Cp	1*Res	1	48
Ad	1*Ad	1*Res	1	48
CpxAd	1*CpxAd	1*Res	1	48

Estimates of components of variation

Source	Estimate	Sq.root
S(Cp)	9.905	3.1472
S(Ad)	-0.28375	-0.53269
S(CpxAd)	13.225	3.6367
V(Res)	69.136	8.3148

Table 3B – Genus level: abbreviations Factor 1- NE challenge (Cp) and Factor 2 -probiotic additive (Ad)

PERMANOVA

Permutational MANOVA

Resemblance worksheet
Name: Resem1
Data type: Similarity
Selection: All
Resemblance: S17 Bray-Curtis similarity

Sums of squares type: Type III (partial)
Fixed effects sum to zero for mixed terms
Permutation method: Permutation of residuals under a reduced model
Number of permutations: 99999

Factors

Name	Abbrev.	Type	Levels
Cp	Cp	Fixed	2
Aditive	Ad	Fixed	2

PERMANOVA table of results

Source	df	SS	MS	Pseudo-F	P(perm)	Unique perms	P(MC)
Cp	1	456.67	456.67	1.987	0.064	93875	0.071
Ad	1	404.26	404.26	1.759	0.0981	93880	0.1043
CpxAd	1	316.31	316.31	1.3763	0.1969	93966	0.2008
Res	48	11032	229.83				
Total	51	12202					

Details of the expected mean squares (EMS) for the model

Source	EMS
Cp	1*V(Res) + 25.923*S(Cp)
Ad	1*V(Res) + 25.923*S(Ad)
CpxAd	1*V(Res) + 12.961*S(CpxAd)
Res	1*V(Res)

Construction of Pseudo-F ratio(s) from mean squares

Source	Numerator	Denominator	Num.df	Den.df
Cp	1*Cp	1*Res	1	48
Ad	1*Ad	1*Res	1	48
CpxAd	1*CpxAd	1*Res	1	48

Estimates of components of variation

Source	Estimate	Sq.root
S(Cp)	8.7508	2.9582
S(Ad)	6.729	2.594
S(CpxAd)	6.6719	2.583
V(Res)	229.83	15.16

Table 3C – an OTU level: abbreviations Factor 1- NE challenge (Cp) and Factor 2 -probiotic additive (Ad)

PERMANOVA

Permutational MANOVA

Resemblance worksheet

Name: Resem3

Data type: Similarity

Selection: All

Resemblance: S17 Bray-Curtis similarity

Sums of squares type: Type III (partial)

Fixed effects sum to zero for mixed terms

Permutation method: Permutation of residuals under a reduced model

Number of permutations: 99999

Factors

Name	Abbrev.	Type	Levels
Cp	Cp	Fixed	2
Aditive	Ad	Fixed	2

PERMANOVA table of results

Source	df	SS	MS	Pseudo-F	P(perm)	Unique perms	P(MC)
Cp	1	2562.2	2562.2	1.7576	0.0079	87709	0.023
Ad	1	2012.1	2012.1	1.3802	0.0651	87697	0.1088
CpxAd	1	2181.3	2181.3	1.4963	0.032	87667	0.069
Res	48	69976	1457.8				
Total	51	76725					

Details of the expected mean squares (EMS) for the model

Source	EMS
Cp	1*V(Res) + 25.923*S(Cp)
Ad	1*V(Res) + 25.923*S(Ad)
CpxAd	1*V(Res) + 12.961*S(CpxAd)
Res	1*V(Res)

Construction of Pseudo-F ratio(s) from mean squares

Source	Numerator	Denominator	Num.df	Den.df
Cp	1*Cp	1*Res	1	48
Ad	1*Ad	1*Res	1	48
CpxAd	1*CpxAd	1*Res	1	48

Estimates of components of variation

Source	Estimate	Sq.root
S(Cp)	42.604	6.5271
S(Ad)	21.382	4.624
S(CpxAd)	55.819	7.4712
V(Res)	1457.8	38.182
