# Supplementary material for: Bacillus subtilis PB6 based probiotic supplementation plays a role in the recovery after the necrotic enteritis challenge
Source: PLoS One. 2020 Jun 18;15(6):e0232781. doi: 10.1371/journal.pone.0232781 (PMC7302482; doi:10.1371/journal.pone.0232781)
Supplement: S1 Fig — (PDF) [file pone.0232781.s004.pdf]

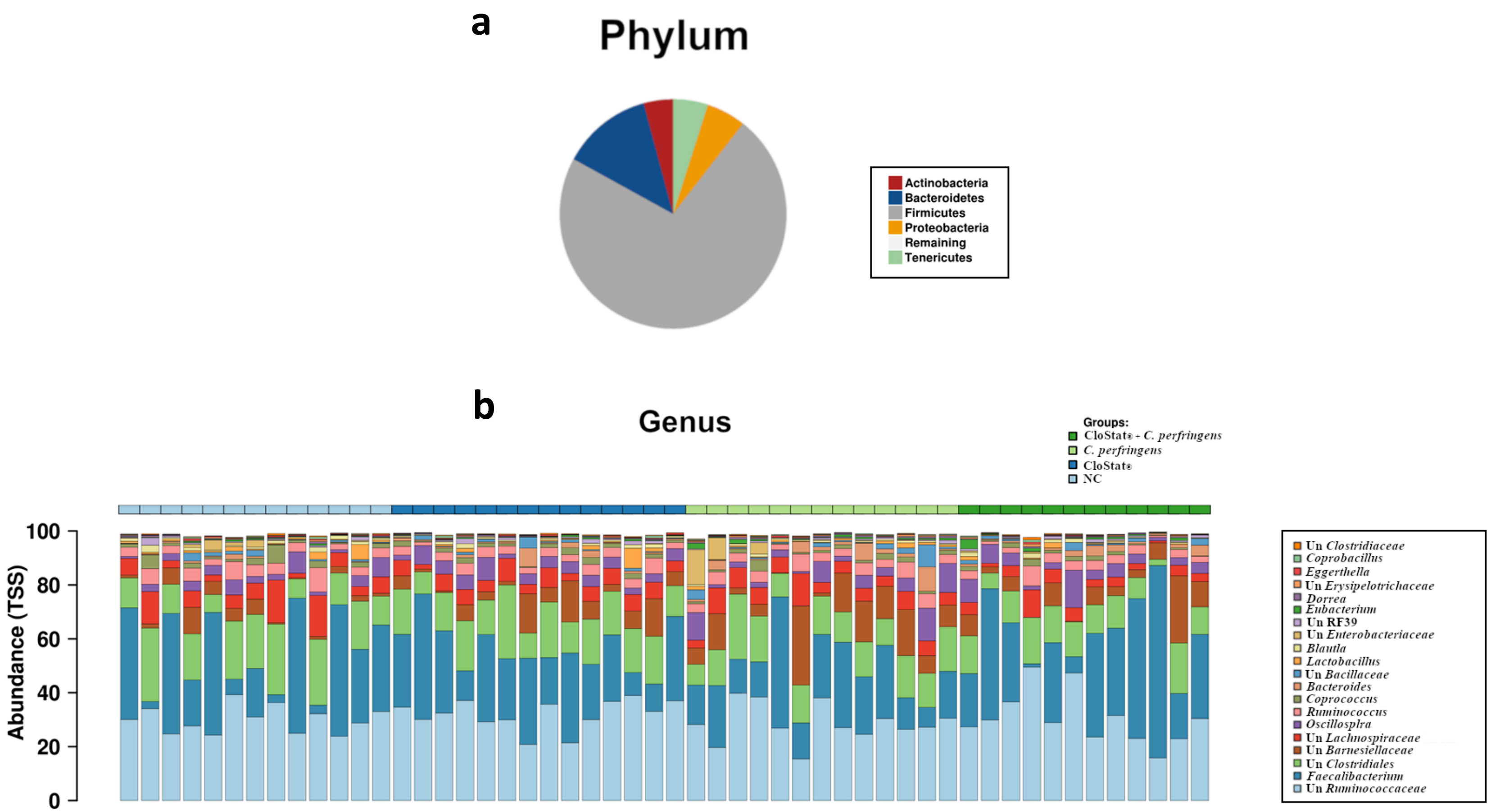

Fig. S1: Cecal microbiota composition of broilers within different groups; (A) the most dominant phyla, and (B) the 20 most abundant genera.
