# Supplementary material for: Bacillus subtilis PB6 based probiotic supplementation plays a role in the recovery after the necrotic enteritis challenge
Source: PLoS One. 2020 Jun 18;15(6):e0232781. doi: 10.1371/journal.pone.0232781 (PMC7302482; doi:10.1371/journal.pone.0232781)
Supplement: S2 Fig — (PDF) [file pone.0232781.s005.pdf]

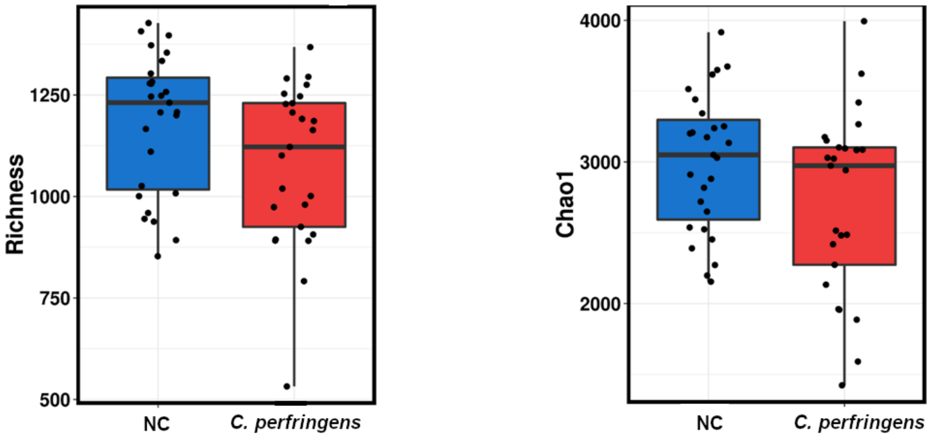

Fig. S2: Impact of challenge on *C. perfringens* impact on Alpha diversity, lower Richness in association with challenge (P = 0.047) (left) marginally influenced Chao1 index (P = 0.089) (right), NC: non- challenged.
