# Supplementary material for: Bacillus subtilis PB6 based probiotic supplementation plays a role in the recovery after the necrotic enteritis challenge
Source: PLoS One. 2020 Jun 18;15(6):e0232781. doi: 10.1371/journal.pone.0232781 (PMC7302482; doi:10.1371/journal.pone.0232781)
Supplement: S3 Fig — (PDF) [file pone.0232781.s006.pdf]

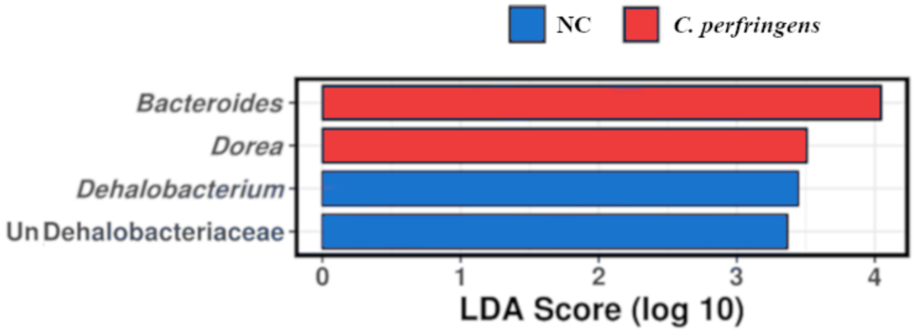

Fig. S3: Linear discriminant analysis and effect size method (LEfSe) showing the genera that is most likely to describe the differences between groups.
